# Loss of SIRT2 leads to axonal degeneration and locomotor disability associated with redox and energy imbalance

**DOI:** 10.1111/acel.12682

**Published:** 2017-10-05

**Authors:** Stéphane Fourcade, Laia Morató, Janani Parameswaran, Montserrat Ruiz, Tatiana Ruiz‐Cortés, Mariona Jové, Alba Naudí, Paloma Martínez‐Redondo, Mara Dierssen, Isidre Ferrer, Francesc Villarroya, Reinald Pamplona, Alejandro Vaquero, Manel Portero‐Otín, Aurora Pujol

**Affiliations:** ^1^ Neurometabolic Diseases Laboratory Institute of Neuropathology IDIBELL Barcelona Spain; ^2^ CIBERER U759 Center for Biomedical Research on Rare Diseases Barcelona Spain; ^3^ Biogenesis Research Group Agrarian Sciences Faculty University of Antioquia Medellin Colombia; ^4^ Experimental Medicine Department University of Lleida‐IRBLleida Lleida Spain; ^5^ Chromatin Biology Laboratory, Cancer Epigenetics and Biology Program (PEBC) Bellvitge Biomedical Research Institute (IDIBELL) 08908 L'Hospitalet de Llobregat, Barcelona Spain; ^6^ Cellular & Systems Neurobiology, Systems Biology Program, Centre for Genomic Regulation The Barcelona Institute of Science and Technology Barcelona Spain; ^7^ Department of Experimental and Health Sciences Universidad Pompeu Fabra Barcelona Spain; ^8^ CIBERER U716 Center for Biomedical Research on Rare Diseases Barcelona Spain; ^9^ Institute of Neuropathology University of Barcelona L'Hospitalet de Llobregat, Barcelona Spain; ^10^ Center for Biomedical Research on Neurodegenerative Diseases (CIBERNED) ISCIII Madrid Spain; ^11^ Department of Biochemistry and Molecular Biology University of Barcelona Av. Diagonal 643 08028 Barcelona, Catalonia Spain; ^12^ The Institute of Biomedicine of the University of Barcelona (IBUB) Barcelona Spain; ^13^ Center for Biomedical Research on Physiopathology of Obesity and Nutrition (CIBEROBN) Barcelona Spain; ^14^ Catalan Institution of Research and Advanced Studies (ICREA) Barcelona Spain

**Keywords:** aging, axonal degeneration, mitochondria, redox dyshomeostasis, sirtuin

## Abstract

Sirtuin 2 (SIRT2) is a member of a family of NAD ^+^‐dependent histone deacetylases (HDAC) that play diverse roles in cellular metabolism and especially for aging process. SIRT2 is located in the nucleus, cytoplasm, and mitochondria, is highly expressed in the central nervous system (CNS), and has been reported to regulate a variety of processes including oxidative stress, genome integrity, and myelination. However, little is known about the role of SIRT2 in the nervous system specifically during aging. Here, we show that middle‐aged, 13‐month‐old mice lacking SIRT2 exhibit locomotor dysfunction due to axonal degeneration, which was not present in young SIRT2 mice. In addition, these *Sirt2* ^−/−^ mice exhibit mitochondrial depletion resulting in energy failure, and redox dyshomeostasis. Our results provide a novel link between SIRT2 and physiological aging impacting the axonal compartment of the central nervous system, while supporting a major role for SIRT2 in orchestrating its metabolic regulation. This underscores the value of SIRT2 as a therapeutic target in the most prevalent neurodegenerative diseases that undergo with axonal degeneration associated with redox and energetic dyshomeostasis.

## Introduction

Age‐related neurodegenerative diseases are a large group of debilitating pathologies that includes Alzheimer's disease (AD), Parkinson's disease (PD), Huntington's disease (HD), and multiple sclerosis (MS). They are characterized by progressive neuronal degeneration, which leads to cell death (McSharry, 2010). Many of these diseases share common pathogenetic mechanisms, including axonal degeneration, which could be responsible for many of the associated motor and cognitive dysfunctions. Other common characteristics underlying many age‐related, multifactorial neurodegenerative diseases are chronic impairment of bioenergetics and mitochondria metabolism, together with redox dyshomeostasis, which cause defects in protein degradation and inflammation (Lin & Beal, 2006; McWilliams & Ganley, 2016).

Many of the neurodegenerative processes in these disorders have been linked with sirtuin family members (SIRTs) (Donmez & Outeiro, 2013; Morris, 2013). SIRTs, or silent information regulator 2 (Sir2) proteins, are a class of proteins that possess NAD^+^‐dependent deacetylase activity or ADP‐ribosyltransferase activity and play key roles in metabolic adaptive responses. In mammals, among the seven members of the SIRT family (SIRT1–SIRT7), SIRT1 has been the most extensively characterized (Hall *et al*., 2013). SIRTs have different tissue and subcellular localizations (Morris, 2013): SIRT1 and SIRT2 are both localized in the nucleus and cytoplasm; however, SIRT1 is mainly nuclear, whereas SIRT2 is predominantly cytoplasmic. Very recently, it has also been demonstrated that SIRT2 can be located in the inner mitochondrial membrane (Liu *et al*., 2017). SIRT3, SIRT4, and SIRT5 are also mitochondrial proteins, while SIRT6 and SIRT7 are localized in the nucleus. The distinct cellular distributions of the SIRT proteins reflect specialized functions in cellular metabolism (Morris, 2013). For instance, SIRT2 is the most abundant member of the SIRT family in the central nervous system (CNS) (Zhu *et al*., 2012), and its role in these organs has been linked to the regulation of several processes including the oxidative stress response, metabolism, cell differentiation, and mitophagy (Donmez & Outeiro, 2013; Liu *et al*., 2017).

In the CNS, SIRT2 has been shown to play a role in oligodendroglial cell differentiation and myelination (Werner *et al*., 2007; Beirowski *et al*., 2011; Ji *et al*., 2011; Zhu *et al*., 2012). Mice with SIRT2 ablation specifically in Schwann cells displayed a transient delay of myelination both during development and after nerve injury (Beirowski *et al*., 2011). However, whether SIRT2 also impacts axonal degeneration was not analyzed in these mice.

SIRT2 has also been proposed to play a role in neuroinflammation, although this has been controversial. For example, upon inhibition or deletion of SIRT2, stimulation of the immune response by lipopolysaccharide (LPS) led to an overt production of pro‐inflammatory cytokines in an experimental model of colitis and after traumatic brain injury (Lo Sasso *et al*., 2015; Yuan *et al*., 2016), suggesting a role for SIRT2 in inhibiting the inflammatory response. However, a role for SIRT2 in promoting inflammation was found upon LPS treatment in microglial cell lines, macrophages, and mouse brain (Lee *et al*., 2014; Chen *et al*., 2015a; Wang *et al*., 2016). This was also supported by the observed attenuation of cytokine levels in a lethal septic model where the activity of SIRT2 was pharmacologically reduced (Zhao *et al*., 2015). However, ischemic brains of WT and *Sirt2* ^−/−^ mice were characterized by a similar induction of neutrophils and activated microglia/macrophages (Krey *et al*., 2015). Thus, whether and how SIRT2 regulates inflammation in the brain remains unclear.

In addition, several studies have identified a major role for SIRT2 in neurodegeneration (Donmez & Outeiro, 2013; Morris, 2013). Pharmacological and/or genetic inhibition of SIRT2 improves disease pathology in models of AD (Silva *et al*., 2016), HD (Luthi‐Carter *et al*., 2010), PD, and cerebral ischemia, but not in models of the motor neuron disease amyotrophic lateral sclerosis (ALS) (Chen *et al*., 2015b). However, some conflicting results have also emerged in HD, for instance, when lack of SIRT2 had no effect on disease progression specifically in the R6/2 mouse model (Bobrowska *et al*., 2012).

Although these studies indicate that SIRT2 has a major role in the brain, to date there has been no link between SIRT2 and phenotypes related to axonal degeneration or impaired behavioral or cognitive skills in middle‐aged mice. To address this, we here characterize 13‐month‐old *Sirt2* ^−/−^ mice at baseline, performing general behavior experiments (i.e., locomotor tests, circadian activity, anxiety, and cognition), tested immunohistopathological markers of axonal degeneration, and performed biochemical analyses. We discover that ablation of SIRT2 results in axonal degeneration, which was associated with locomotor disability, redox imbalance, and energetic failure. No systemic neuroinflammation or metabolic syndrome was observed. These results suggest that SIRT2 may constitute a valuable therapeutic target in major neurodegenerative diseases associated with aging, which manifest with axonopathy caused by redox and energy metabolism.

## Results

Several studies have revealed a key role for SIRT2 in neurodegenerative (Donmez & Outeiro, 2013) and myelination processes (Werner *et al*., 2007; Beirowski *et al*., 2011; Ji *et al*., 2011; Zhu *et al*., 2012). Primary perturbation of myelinating glial cells has often been linked to profound secondary effects on axonal function. Axonal malfunction is associated with axonal degeneration, which is a common feature and main contributor to incapacity in many neurological diseases such as AD, PD, HD, MS, or adrenomyeloneuropathy (AMN) as well as of aging (Lin & Beal, 2006; Ferrer *et al*., 2010; Galea *et al*., 2012; McWilliams & Ganley, 2016). Therefore, we analyzed whether *Sirt2* ^−/−^ mice develop axonal degeneration in middle‐aged mice (13 months of age).

### *Sirt2* ^−/−^ mice present axonal degeneration at 13 months of age

**Figure 1:**
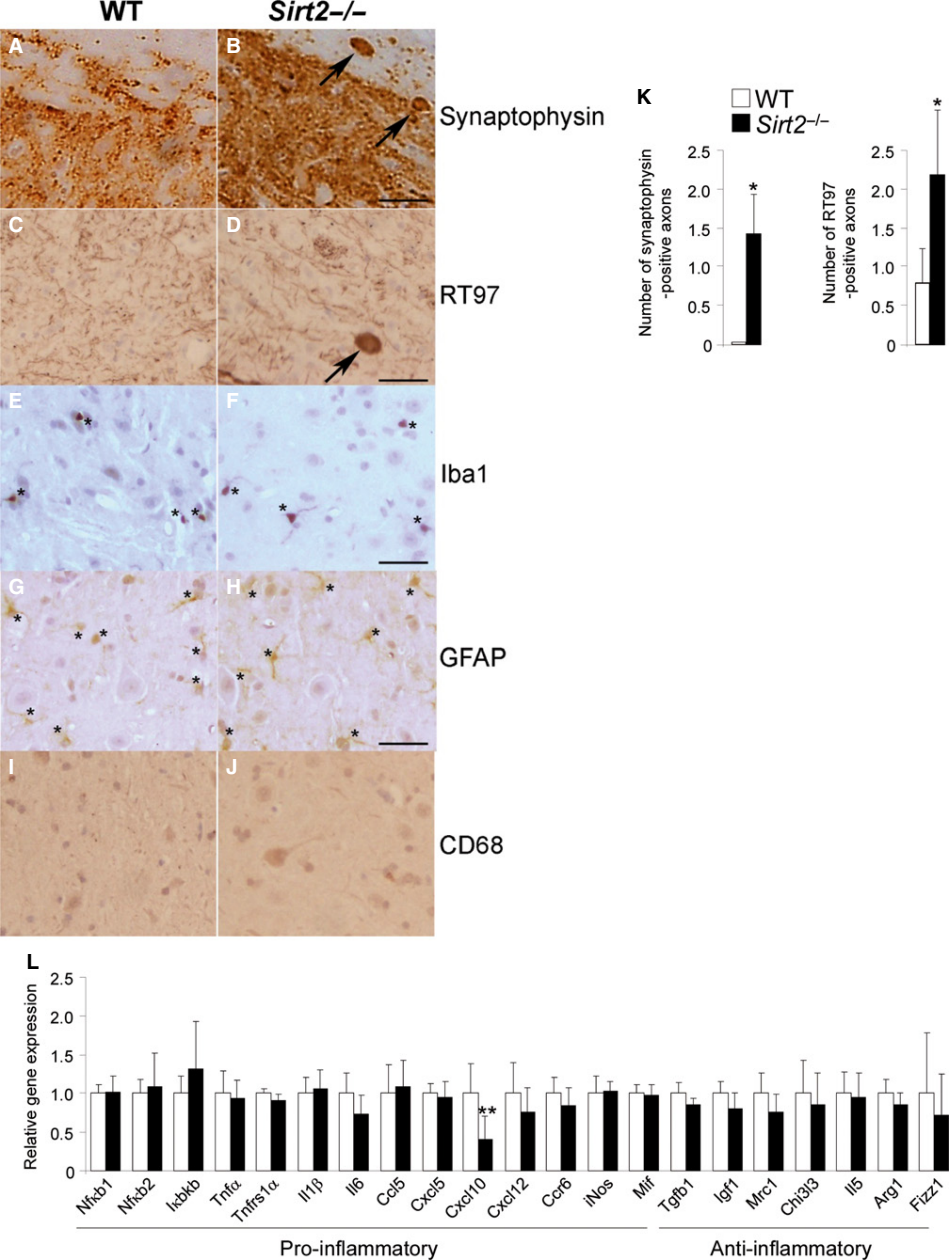
Sirt2^−/−^ mice present axonal degeneration but no neuroinflammation at 13 months of age. Longitudinal and transversal sections of the dorsal spinal cord in WT (A, C, E, G, I) and Sirt2^−/−^ mice (B, D, F, H, J) at 13 months of age (*n* = 5/genotype) processed for synaptophysin (A–B), RT97 (C–D), Iba‐1 (E–F), GFAP (G‐H), and CD68 (I‐J). Arrows indicate synaptophysin (A–B) and RT97 (C–D) accumulation in axonal swellings. Small stars indicate Iba‐1^+^ cells (E‐F) and GFAP ^+^ cells (G‐H). Scale bar = 25 μm. (K) Quantification of synaptophysin and RT97 accumulation in axonal swellings in 1‐cm‐long longitudinal sections of the dorsal spinal cord in WT and Sirt2^−/−^ mice. The number of abnormal specific profiles was counted at every 10 sections for each stain. At least three sections corresponding to the dorsal columns of the spinal cord were analyzed per animal and per stain. (L) Relative mRNA levels of Nfκb1, Nfκb2, Ikbkb, Tnfα, Il1β, Il6, Cxcl10, Cxcl5, Mif, Tnfrs1α, Ccl5, Ccr6, Cxcl12, Tgfβ1, iNos, Igf1, Mrc1, Chi3 l3, Il5, Arg1, and Fizz1 in spinal cord from WT and Sirt2^−/−^ mice at 13 months of age (*n* = 8/genotype). Data represent mean ± SD. Statistical analysis was carried out with Student's *t*‐test (*P ≤ 0.05, **P ≤ 0.01).

We performed immunohistochemistry on spinal cord from middle‐aged *Sirt2* ^−/−^ mice. *Sirt2* ^−/−^ mice presented an overt neuropathological phenotype at 13 months of age characterized by axonal damage revealed by the accumulation of synaptophysin and RT97 in axonal swellings (Fig. 1A–D and K). However, this axonal degeneration was not associated with neuroinflammation in the form of astrocytosis and microgliosis or macrophage infiltration, as revealed by GFAP, Iba‐1, and CD68 staining, respectively (Fig. 1E–J; Morato *et al*., 2015). To support these data, we quantified the expression levels of several cytokines, chemokines, and their receptors in the spinal cord of *Sirt2* ^−/−^ mice. The absence of neuroinflammation in *Sirt2* ^−/−^ mice was consistent with the lack of induction of several pro‐inflammatory molecules as well as the absence of repression of anti‐inflammatory genes. Specifically, genes involved in both the canonical and noncanonical NFκB pathway (*Nfκb1* and *Nfκb2*, respectively) and in the IKK complex (*Ikbkb*), which activates these two pathways, were not dysregulated (Fig. 1L). Our results are the first to analyze the effects on neuroinflammation of SIRT2 at baseline, in the absence of a specific immunological stimulus, and in aging mice. Along with other recently published results (Lee *et al*., 2014; Chen *et al*., 2015a; Krey *et al*., 2015; Lo Sasso *et al*., 2015; Zhao *et al*., 2015; Wang *et al*., 2016; Yuan *et al*., 2016), our results support a strongly context‐dependent and/or indirect role for SIRT2 in the inflammatory response.

### *Sirt2* ^−/−^ mice exhibit locomotor deficits at 13 months of age

**Figure 2:**
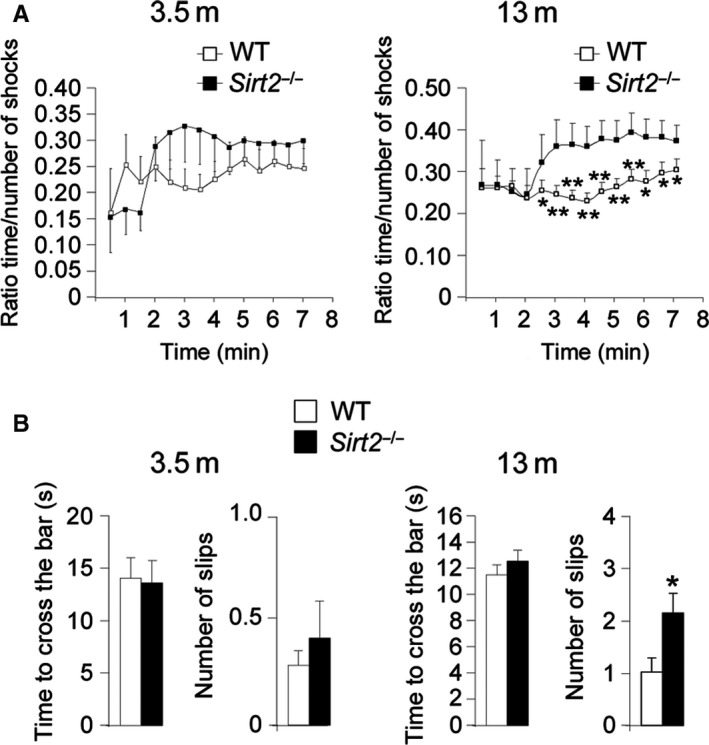
*Sirt2* ^−/−^ mice exhibit locomotor deficits at 13 months of age. (A) Plotted are the ratios between the time length of the shocks (time that mice stay on the electrified grid) and the number of shocks received, over the duration of each test and (B) bar‐cross test in WT and *Sirt2* ^−/−^ mice at 3.5 and 13 months of age (*n* = 20/genotype). No falls were recorded. Data represent mean ± SD. Statistical analysis was carried out with Student's *t*‐test (*P ≤ 0.05, **P ≤ 0.01).

To determine whether the observed axonal degeneration could impact locomotor function, we assessed the performance of *Sirt2* ^−/−^ mice on the treadmill and bar‐cross tests (Morato *et al*., 2015) at 3.5 and 13 months of age. In the treadmill test, where the mice are evaluated by their ability to remain on a moving treadmill, only the oldest *Sirt2* ^−/−^ mice showed a significant locomotor deficit compared to the littermate WT mice (Fig. 2A). There was no difference between the young (3.5 m) *Sirt2* ^−/−^ and WT mice. Also in the bar‐cross experiments, only the 13‐month‐old *Sirt2* ^−/−^ mice failed to maintain their balance while walking across the bar, and displayed a greater tendency to slip off the bar than age‐matched WT mice (Fig. 2B). Thus, *Sirt2* ^−/−^ mice exhibit locomotor disability at 13 but not 3.5 months of age (Fig. 2).

We next performed a general behavioral characterization of middle‐aged *Sirt2* ^−/−^ mice by measuring muscle tone and pain sensitivity (grip strength and hot plate test, respectively), circadian activity, emotional reactivity or anxiety‐like behavior (elevated plus‐maze), and cognition (avoidance learning and novel object recognition). No differences were observed between 13‐month‐old *Sirt2* ^−/−^ mice and control littermates (Figs S1–S3).

Together, our results provide evidences for a role of SIRT2 in axonal degeneration associated with locomotor dysfunction in these mice.

### Redox imbalance in spinal cord from *Sirt2* ^−/−^ mice

**Figure 3:**
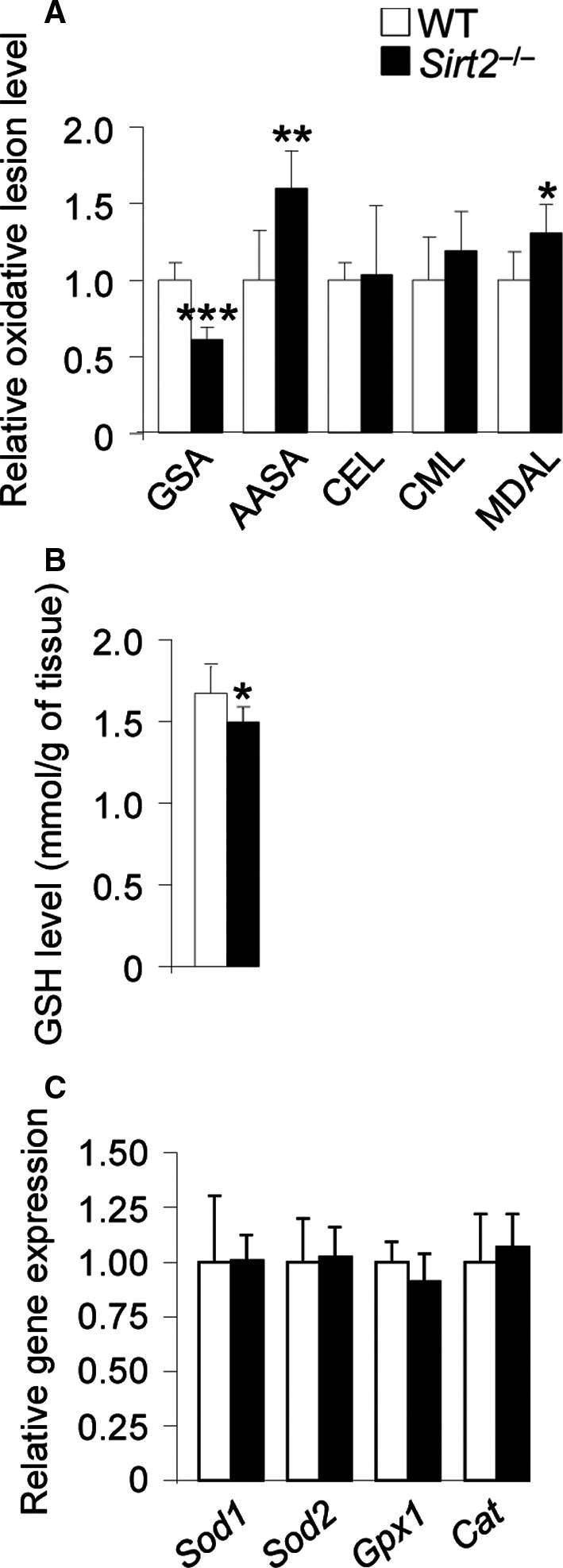
Redox imbalance in spinal cord from *Sirt2* ^−/−^ mice. (A) GSA, AASA, CEL, CML, and MDAL levels and (B) GSH levels and (C) relative mRNA levels of *Sod1*,* Sod2*,* Gpx1*, and *Cat* in spinal cord from WT and *Sirt2* ^−/−^ mice at 13 months of age (*n* = 8/genotype). Data represent mean ± SD. Statistical analysis was carried out with Student's *t*‐test (*P ≤ 0.05, **P ≤ 0.01, ***P ≤ 0.001).

Chronic metabolic impairment and oxidative stress are associated with the pathogenesis of axonal dysfunction in a growing number of neurodegenerative diseases including AD, PD, HD, ALS, AMN, and multiple sclerosis (Lin & Beal, 2006; Ferrer *et al*., 2010; Galea *et al*., 2012). To determine whether the axonal degeneration we observed in *Sirt2* ^−/−^ mice was caused by oxidative stress, we quantified several protein oxidative damage markers including glutamic semialdehyde (GSA) and aminoadipic semialdehyde (AASA) for protein carbonylation, *N* ^ε^‐(carboxymethyl)‐lysine (CML) and *N* ^ε^‐(carboxyethyl)‐lysine (CEL) for glycoxidation, and *N* ^ε^‐(malondialdehyde)‐lysine (MDAL) for lipoxidation (Fourcade *et al*., 2008) (Fig. 3A). We also analyzed reduced glutathione (GSH), which is the main antioxidant molecule in the cell (Fig. 3B). We found increased oxidative damage characterized by higher levels of AASA and MDAL and low level of GSH in spinal cord from 13‐month‐old *Sirt2* ^−/−^ mice compared with control littermates. Further, we measured expression levels of the classical antioxidant enzymes, superoxide dismutase 1 and 2 (*Sod1* and *Sod2*), glutathione peroxidase 1 (*Gpx1*), and catalase (*Cat*), which encode for key enzymes that reduce oxidative stress in cells. mRNA levels of *Sod1*,* Sod2*,* Gpx1*, and *Cat* were unchanged in middle‐aged spinal cord from *Sirt2* ^−/−^ mice (Fig. 3C). This indicates that in the absence of SIRT2, the endogenous antioxidant defense is blunted, causing rising oxidative damage in these mice, which may lead to the observed axonal dysfunction.

### mtDNA depletion and energy failure in *Sirt2* ^−/−^ mice

**Figure 4:**
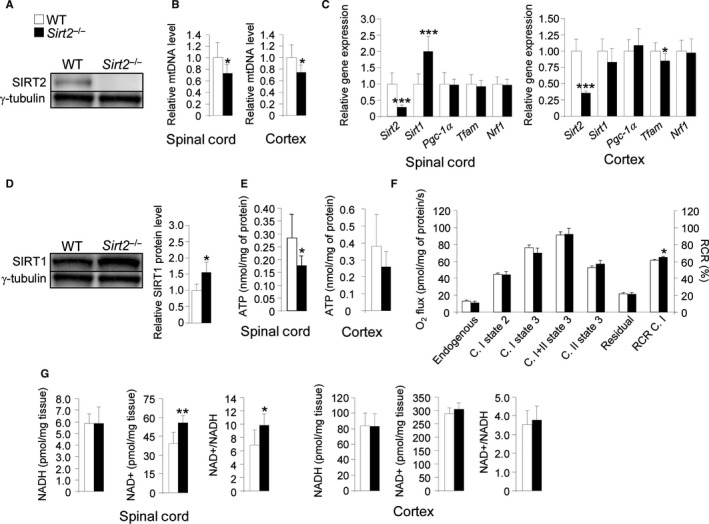
mtDNA depletion and energy failure in *Sirt2* ^−/−^ mice. (A) SIRT2 protein level in spinal cord from WT and *Sirt2* ^−/−^ mice at 13 months of age (*n* = 6/genotype), (B) mtDNA levels in spinal cord and cortex (*n* = 8/genotype), (C) relative mRNA levels of *Sirt2*,* Sirt1*,* Pgc‐1α*,* Tfam*, and *Nrf1* in spinal cord and cortex (*n* = 8/genotype), (D) SIRT1 protein level in spinal cord (*n* = 6/genotype), (E) ATP levels in spinal cord and cortex (*n* = 8/genotype), (F) *ex vivo* mitochondrial respiration analysis performed on permeabilized sections of spinal cord (*n* = 5/genotype), and (G) NAD ^+^, NADH, and NAD ^+^/NADH ratio in spinal cord and cortex (*n* = 8/genotype) from WT and *Sirt2* ^−/−^ mice at 13 months of age. Data represent mean ± SD. Statistical analysis was carried out with Student's *t*‐test (*P ≤ 0.05, **P ≤ 0.01, ***P ≤ 0.001).

To study the global consequences of altered redox status, and because sirtuins are associated with mitochondrial function and metabolism, we quantified mtDNA levels as well as ATP, NAD^+^, and NADH levels in *Sirt2* ^−/−^ mice. We have observed no SIRT2 protein expression in spinal cord from *Sirt2* ^−/−^ mice, which validates that this model is a true knockout of SIRT2 (Fig. 4A). MtDNA levels were lower in both spinal cord and cortex of *Sirt2* ^−/−^ mice at 13 months compared to control littermates (Fig. 4B). Of note, this depletion did not appear to be mediated by the main master regulator of mitochondrial biogenesis *Pgc‐1α* and its targets *Tfam* and *Nrf1* (Scarpulla *et al*., 2011) as their expression levels did not vary (Fig. 4B), although we cannot rule out other means of controlling Sirt2 expression. We also observed an increase in expression levels of SIRT1 (at both protein and mRNA levels) in spinal cord (Fig. 4C,D). Because SIRT1 upregulates PGC‐1α, this may be a compensatory mechanism to prevent mitochondrial depletion in the absence of SIRT2.

We next quantified ATP levels in spinal cord from *Sirt2* ^−/−^ mice (Fig. 4E). ATP levels were significantly reduced in *Sirt2* ^−/−^ mice, which correlated with an increase in the respiratory control ratio (RCR C. I) (ratio: complex I (C. I) state 2/C. I state 3) (Fig. 4F). Further, we found that NAD^+^ but not NADH was elevated in spinal cord from *Sirt2* ^−/−^ mice (Fig. 4G). Given that NAD^+^ is the limiting substrate for sirtuin activities, and *Sirt2* is highly expressed in spinal cord, our results suggests that SIRT2 could be the main NAD^+^ consumer in spinal cord from the sirtuin family.

### No systemic metabolic syndrome in *Sirt2* ^−/−^ mice

**Figure 5:**
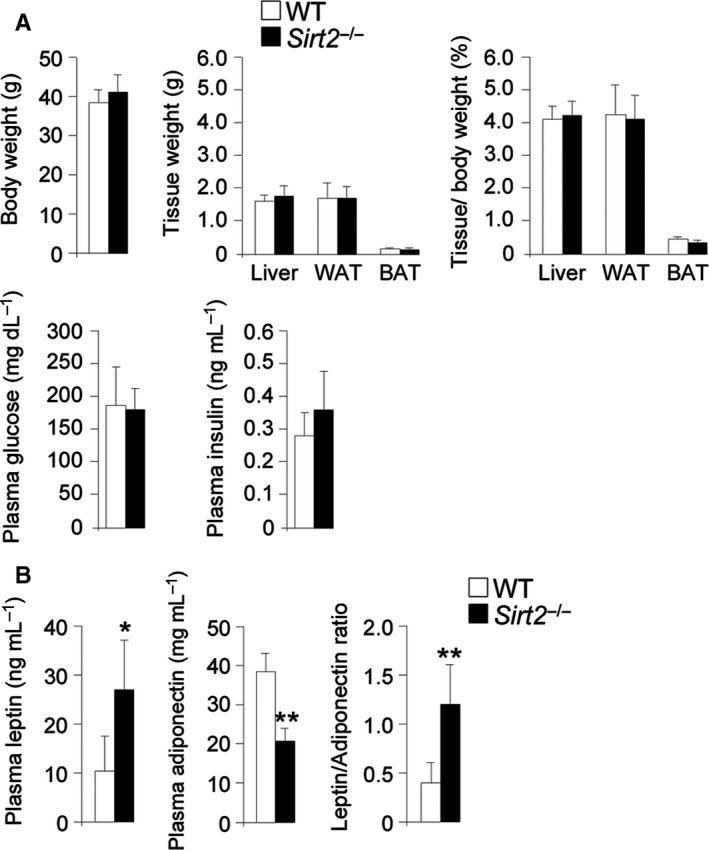
No metabolic syndrome in *Sirt2* ^−/−^ mice. (A) Body and tissue (liver, WAT, BAT) weight, tissue weight/body weight ratio, and glucose and insulin plasma levels in WT and *Sirt2* ^−/−^ mice at 13 months of age (*n* = 6/genotype). (B) Adiponectin, leptin, and leptin/adiponectin ratio in blood from WT and *Sirt2* ^−/−^ mice at 13 months of age (*n* = 6/genotype). Data represent mean ± SD. Statistical analysis was carried out with Student's *t*‐test (*P ≤ 0.05, **P ≤ 0.01).

Given the observed effect of SIRT2 loss on redox and energy imbalance, we quantified metabolically relevant parameters in the 13‐month‐old *Sirt2* ^−/−^ mice, including body and tissue weight, and glucose and insulin levels in plasma. No differences were observed compared to WT (Fig. 5A). Similar data have been reported for 3.5‐month‐old mice (Belman *et al*., 2015). To gain further insight into any adipocyte‐related dysregulation, we measured the levels of leptin and adiponectin, which are the most abundant adipocytokines produced by adipocytes. We found that adiponectin levels were reduced, whereas leptin was increased in serum from *Sirt2* ^−/−^ mice at 13 months of age with respect to controls (Fig. 5B). Altogether, these results indicate that both young and middle‐aged *Sirt2* ^−/−^ mice do not develop a systemic metabolic syndrome or insulin resistance at baseline, in spite of the noted imbalance of adiponectin and leptin levels.

## Discussion

### SIRT2 and axonal degeneration

In the current study, we revealed for the first time that middle‐aged *Sirt2* ^−/−^ mice exhibited axonal degeneration associated with locomotor disability in the absence of cognitive malfunction (Figs 1 and 2, Figs S1–S3). This may suggest a role for SIRT2 in the axonal degeneration cascade of neurodegenerative disorders of aging such as MS (McSharry, 2010), ALS (Fischer‐Hayes *et al*., 2013), X‐linked adrenomyeloneuropathy (X‐AMN) (Fourcade *et al*., 2015), or the hereditary spastic paraplegias (Fink, 2013). This is supported by two other studies in which SIRT2 was reduced, both in a model of spastic paraplegia SPG2 (Werner *et al*., 2007) and in a model of long‐term vitamin E‐deficient mice (Fukui *et al*., 2012). The SPG2 mice lack proteolipid protein 1 (PLP) and develop axonal degeneration (Griffiths *et al*., 1998). Subsequently, PLP was found to be involved in the transport of SIRT2 and is abundant in myelin of the CNS (Werner *et al*., 2007; Zhu *et al*., 2012). Thereby, the lack of PLP led to decreased levels of SIRT2 in the brain (Werner *et al*., 2007). In the mouse model of vitamin E deficiency, axonal degeneration appeared in association with reduced SIRT2 levels in several areas of the brain (cortex, cerebellum, and hippocampus) at 6 months of age. In the same model, SIRT2 levels were unchanged at 3 months of age, when no axonal degeneration was observed (Fukui *et al*., 2012).

### SIRT2 and neuroinflammation

No signs of neuroinflammation have been observed at steady‐state level in the spinal cord of middle‐aged *Sirt2* ^−/−^ mice when using markers of astrogliosis (GFAP) or microgliosis (Iba‐1) (Fig. 1E–H), corroborated by lack of activation of pro‐inflammatory cytokines (Fig. 1L). We believe that the lack of neuroinflammation could be attributed to a compensatory upregulation of SIRT1 in the spinal cords in this model. Indeed, we have previously reported that genetic or pharmacological induction (by resveratrol) of SIRT1 suppressed microgliosis and astrogliosis in a mouse model of X‐ALD (Morato *et al*., 2015).

### SIRT2 and redox/energy homeostasis

In the present work, we identified a pro‐oxidative status in 13‐month‐old Sirt2^−/−^ mice based on the levels of nonenzymatic protein damage markers and the content of general antioxidant defenses (Fig. 3). Thus, 13‐month‐old *Sirt2* ^−/−^ mice did show increased oxidative (AASA) and lipoxidative (MDAL) damage, with no changes for glycoxidative markers (CEL and CML), suggesting a preferential type of protein damage, at least at this age. Surprisingly, we found dissociation between the protein carbonyls AASA (increased) and GSA (decreased). This discordance could be ascribed to the residues which act as substrates for its formation: Arg/Pro residues for GSA and Lys residues for AASA. In this context, the clear observed divergence for the content of AASA vs. GSA suggests differences in the proteomic profile of *Sirt2* ^−/−^ vs. wild‐type mice leading to the presence of a protein pool which differs in structural and functional properties (amino acids profile, location in flexible regions, solvent accessibility, proximity with possible metal ion binding sites) prone to a specific type of damage. Further studies, probably in the context of proteomic analysis, are, however, needed to confirm this hypothesis. Along these lines, lower levels of GSH were detected in *Sirt2* ^−/−^ mice contributing to generate a pro‐oxidative status at this age. The impaired redox status appears not to be restricted to spinal cords, as Liu *et al*. showed an increase in the GSSH/GSH ratio in striatum (Liu *et al*., 2017). However, this was associated with an overall increased ratio of GSSG/GSH, which indicates a pro‐oxidative status (Liu *et al*., 2017). In addition, we showed that SIRT2 regulated energy homeostasis (ATP content and NAD^+^ levels) in the CNS of middle‐aged *Sirt2* ^−/−^ mice (Fig. 4), which is supported by data for ATP in both young and aged mice (Liu *et al*., 2017). A role for SIRT2 in redox status and energy homeostasis has been suggested in several studies due to its capacity to regulate the activities of specific transcription factors and metabolic enzymes. For example, SIRT2 deacetylated and thus activated FOXO3a and its target genes such as *Sod2*, which is a key player in endogenous antioxidant defense. In the absence of SIRT2, SOD2 was not activated (Wang *et al*., 2007). In addition, SIRT2 regenerated GSH levels *via* the conversion of NADP^+^ to NADPH, under oxidative stress conditions (Wang *et al*., 2014). Regarding NADPH production, SIRT2 has been shown to deacetylate and thereby activate glucose‐6‐phosphate dehydrogenase (G6PD), the first enzyme of the pentose phosphate pathway (PPP) (Wang *et al*., 2014), and the glycolytic enzyme phosphoglycerate mutase (PGAM) (Xu *et al*., 2014). PGAM regulates NADPH homeostasis because both substrate (3‐phosphoglycerate) and product (2‐phosphoglycerate) are allosteric regulators of the PPP (Hitosugi *et al*., 2012). In the absence of SIRT2, G6PD and PGAM were degraded and NADPH levels lowered. In conclusion, SIRT2 is thought to control the PPP switch, and therefore NADPH and GSH pools (Wang *et al*., 2014; Xu *et al*., 2014).

With respect to energy homeostasis, it has been shown that the inhibition of SIRT2 in both neuron‐like PC12 (Nie *et al*., 2011) and microglia BV2 cell lines (Li *et al*., 2013; Nie *et al*., 2014) resulted in ATP reduction followed by cell death. In the absence of SIRT2, we find NAD^+^ levels increase in spinal cord. As NAD^+^ is a substrate of poly(ADP‐ribose) polymerase (PARP1) (Rack *et al*., 2016), we speculate that PARP1 could be activated in the CNS of *Sirt2* ^−/−^ mice. Consistent with this, cell death in BV2 microglial cell lines after SIRT2 inhibition depended on PARP1 activation (Li *et al*., 2013).

### SIRT2 and the metabolic syndrome

In our study, middle‐aged *Sirt2* ^−/−^ mice did not develop a systemic metabolic syndrome/insulin resistance (Fig. 5). This is consistent with several experiments in young adult *Sirt2* ^−/−^ mice: 3.5‐month‐old *Sirt2* ^−/−^ mice had lower blood glucose levels compared with control mice (Belman *et al*., 2015). Further, it was demonstrated that SIRT2 regulates PECK1 expression, which is the limiting enzyme for gluconeogenesis. Elevated gluconeogenesis is associated with type II diabetes. The authors showed that reduction of SIRT2, using adenovirus containing shRNA against SIRT2 in mice, resulted in degradation of PECK1 in the liver and decreased glucose levels in the blood (Jiang *et al*., 2011). However, SIRT2 has been linked with metabolic syndrome/insulin resistance under different challenges. Under high‐fat conditions, WT mice gained more weight compared to mice where HIF1α was deleted specifically in white adipose tissue (WAT), indicating a protective role for HIF1α against obesity. The underlying molecular mechanism was found to be dependent on SIRT2: HIF1α bound to the SIRT2 promoter and decreased its expression. Further, in these *Hif1*α knockout mice, PGC‐1α was not activated by SIRT2, which decreased mitochondria numbers, energy expenditure, and fatty acid catabolism. To corroborate this role for HIF1α and SIRT2 in obesity, their expression levels were quantified in visceral white adipose tissue of human obese individuals. Consistently, HIF1α was induced, whereas SIRT2 was reduced in obese patients (Krishnan *et al*., 2012).

### SIRT2 and aging

Aging is defined by nine main hallmarks: genomic instability, telomere attrition, epigenetic alterations, loss of proteostasis, deregulated nutrient sensing, mitochondrial dysfunction, cellular senescence, stem cell exhaustion, and altered intercellular communication (Lopez‐Otin *et al*., 2016). In the present study, we find that middle‐aged *Sirt2* ^−/−^ mice exhibit mitochondrial depletion, redox and energetic imbalance, and axonal degeneration. Previous work has shown that SIRT2 induces the checkpoint kinase BubR1 to increase lifespan in mice (North *et al*., 2014), and revealed SIRT2 to be a novel marker of cellular senescence (Anwar *et al*., 2016). In addition, other studies have linked SIRT2 with DNA damage and genome instability, epigenetic regulation, and stem cell differentiation (Serrano *et al*., 2013; Stein & Imai, 2014). Our data reinforce the notion of SIRT2 acting as a powerful regulator of aging with a potential role in neurodegeneration. Indeed, pathology of aging could appear earlier in rodents (middle‐aged) as already observed in mouse models of several age‐related disorders such as AD or ALS.

## Materials and methods

### Mouse strain

The generation and genotyping of *Sirt2* ^−/−^ mice have been previously described (Serrano *et al*., 2013). We did not observe SIRT2 protein expression in spinal cord from *Sirt2* ^−/−^ mice (Fig. 4A). The mice used for the study were of a pure C57BL/6J background. The animals were sacrificed and the tissues were recovered and stored at −80 °C. All of the methods employed in this study were in accordance with the Guide for the Care and Use of Laboratory Animals published by the U.S. National Institutes of Health (NIH Publications No. 85‐23, revised 1996) and the guidelines of the ethical committees of IDIBELL and the Generalitat de Catalunya, Spain.

### Locomotor tests

Locomotor function was performed in WT and *Sirt2* ^−/−^ mice at 3.5 and 13 months of age under blinded conditions as described (Pujol *et al*., 2004; Morato *et al*., 2015).

### Circadian activity (CA)

CA was measured using activity cages (TSE, Germany) that consist of infrared detection photobeams for analysis of horizontal activity. The test was performed under low‐nonaversive lighting conditions (50 lux.) to avoid stressful stimuli. Total distance travelled each 30 min was recorded for 24 h. Mice were individually housed in standard Makrolon cages (40 cm long × 25 cm wide × 20 cm high) during 24 h for 2 consecutive days.

### Muscle tone and pain sensitivity

#### Grip strength

To assess forelimb and hindlimb strength, mice were held by the tail and placed on the apparatus so that they grabbed the handle with both front paws and then were gently pulled back until they released it. Each session consisted of three trials.

#### Hot plate

To assess nociception, mice were placed on a hot plate at 52 ± 0.1 °C surrounded by a plastic cylinder (19 cm diameter, 19 cm high), and latency to paw licking and jump was manually registered by the experimenter. A maximum latency of 300 s was set to avoid tissue damage.

### Emotional reactivity

To study anxiety‐like behavior, mice were placed on an elevated plus‐maze that consisted of a black Plexiglas apparatus with four arms (29 cm long × 5 cm wide) set in cross from a neutral square (5 × 5 cm). Two opposite arms were delimited by vertical walls (closed arms), and the other two arms had unprotected edges (open arms). The maze was elevated 40 cm above the floor and placed under indirect light (100 lx.). At the beginning of a 5‐min session, each mouse was placed in the central zone, facing one of the open arms. A video‐tracking camera registered time spent, number of entries, speed, and distance in the different zones.

### Cognition

#### Avoidance learning

This test assessed the formation of recent and long‐term memory (consolidation) using a negative reinforcement, and has strong dependence on the cholinergic system. The passive avoidance paradigms consist of a circular platform (3 cm diameter) located on the center of an electrified grid. Mice were placed on the small platform, and when they stepped down, they received a foot shock (0.6 mA; 2 s). The latency to step down from the platform was automatically registered by the apparatus until a maximum of 300 s. On day 1, mice were trained with the negative reinforcement; 24 h later, they were tested; and 1 week later, they were retested for avoidance learning.

#### Novel object recognition

This test evaluates recognition memory for previously explored objects, a cognitive domain that depends on hippocampal functional integrity. Novel object recognition was examined in a Y‐maze apparatus consisting of three adjacent arms (each arm is 30 cm × 5 cm × 6 cm) delineating a Y shape, and thus restricting the zone where mice are allowed to move, increasing the amount of exploration in all groups of mice. The protocol consisted of three sessions:

1. *Habituation session* Animals were habituated for 8 min in the Y‐maze. During this habituation session, the number of entries in the three arms was manually recorded by the experimenter. Moreover, the percentage of spontaneous alternation was also measured. Spontaneous alternation was considered when mice consecutively entered nonpreviously explored arms. The percentage of spontaneous alternation was mathematically estimated as the number of consecutive entrances divided by the maximum alternation (total number of entries divided by 3) and ×100.
2. *Familiarization session* Four hours later, mice were placed in the Y‐maze that contained two identical objects, plastic‐made, and located on two of the arm ends. All mice were placed on the end of the arm that did not contain any object. Time exploring the two objects was recorded for 8 min.
3. *Test session* After 24 h, mice will be presented to two objects for 8 min; one will be the same used in the familiarization session and the other will be a novel one. The discrimination index is calculated as time exploring the novel object – time exploring the familiar object/total time of exploration × 100. The position of the novel object will be counterbalanced between animals.

The arena and objects were deeply cleaned between animals to avoid olfactory cues. All measures of exploration were registered manually by the experimenter blind to genotype or treatment. Exploratory behavior is defined as the animal directing its nose toward the object at a distance of < 2 cm. Sitting on or resting against the object was not considered as exploration.

### RNA and DNA extraction

Total RNA was extracted using RNeasy Kit (Qiagen) according to the manufacturer's instructions. Total DNA was extracted using Gentra Puregene Tissue Kit (Qiagen) according to the manufacturer's instructions.

### Quantitative real‐time PCR

TaqMan real‐time PCR was performed in the LightCycler^®^ 480 Real‐Time PCR System (Roche Diagnostics GmbH, Mannheim, Baden‐Württemberg, Germany) using the TaqMan Universal PCR master mix and the standardized probes for mouse: Arg1 (Mm00475991_m1), Cat (Mm00437992_m1), Ccl5 (Mm01302427_m1), Ccr6 (Mm99999114_s1), Chi3 l3/Ym1 (Mm00657889_mH), Cxcl10 (Mm00445235_m1), Cxcl5 (Mm00436451_m1), Fizz1/Retnla (Mm00445109_m1), Gpx1 (Mm00656767_g1), Ikbkb (Mm01222247_m1), Il1β (Mm01336189_m1), Il5 (Mm00439646_m1), Il6 (Mm00446190_m1), Mif_LDC (Mm 01611157_gH), Mrc1 (Mm01329362_m1), Nfkb1 (Mm00476379_m1), Nfkb2 (Mm00479807_m1), Nos2 (Mm00440502_m1), Nrf‐1 (Mm00447996), Pgc‐1α (Mm00447183_m1), Sirt1 (Mm00490758_m1), Sod1 (Mm01700393_g1), Sod2 (Mm00449726_m1) Tfam (Mm00447485), Tgfβ1 (Mm01178820_m1), Tnfα (Mm00443258_m1), and Tnfrsf1 (Mm01182929_m1). To quantify mouse mtDNA content, mouse cytochrome b (cytb) probe has been designed (Custom TaqMan Gene Expression Assays; Applied Biosystems). The sequences for mouse cytb were ATGACCCCAATACGCAAAATTA (forward) and GGAGGACATAGCCTATGAAGG (reverse), and the FAM‐labeled probe was TTGCAACTATAGCAACAG. Quantification of mtDNA was referred to nuclear DNA as determined by the amplification of the mouse intronless nuclear gene C/EBPα (Mm00514283). Expression of the genes of interest was normalized to that of the reference control mouse Rpl0 (Mm01974474_gH). Each sample was run in duplicate, and the mean value of the duplicate was used to calculate the mtDNA amount or mRNA expression using the comparative (2^−ΔCt^) method, according to the manufacturer's instructions (Morato *et al*., 2015).

### Glucose, insulin, leptin, and adiponectin levels

Glucose was measured in blood using the OneTouch Ultra Easy glucose meter. Insulin, leptin, and adiponectin were quantified in plasma with ELISA kits from BioVendor (RAI005R, #RD291001200R, #RD293023100R) according to the manufacturer's instructions.

### GSH levels, NAD^+^/NADH determination, and ATP levels

The reduced glutathione (GSH) levels were determined by LC‐ESI‐qTOF‐MS as previously described (Morato *et al*., 2015). NAD^+^/NADH levels were measured by the NAD cycling assay. The ATP levels were measured by a chemiluminescence system using ATPlite 1step (Perkin Elmer) (Morato *et al*., 2015).

### Measurement of GSA (glutamic semialdehyde), AASA (aminoadipic semialdehyde), CEL (*N* ^*∈*^‐(carboxyethyl)‐lysine), CML (*N* ^*∈*^‐(carboxymethyl)‐lysine), and MDAL (*N* ^*∈*^‐malondialdehyde‐lysine)

GSA, AASA, CEL, CML, and MDAL concentrations in total proteins from spinal cord homogenates were measured by gas chromatography/mass spectrometry (GC/MS) (Fourcade *et al*., 2008). The product levels were expressed as the ratio of micromoles of GSA, AASA, CEL, CML, or MDAL to 1 mole of lysine.

### Immunoblot analysis

Tissue samples were lysed in ice‐cold RIPA buffer (50 mm Tris–HCl, pH 8, 12 mM deoxycholic acid, 150 mm NaCl, and 1% NP‐40, supplemented with Complete Protease Inhibitor Cocktail (Roche) and Phosphatase Inhibitor Cocktail ‘PhosSTOP EASYpack’ (Roche)) using a Teflon‐on‐glass homogenizer and then centrifuged at 1500 *g* for 10 min at 4 °C. The protein concentration was determined using a BCA protein assay kit (Thermo Fisher Scientific, Inc., Waltham, Massachusetts, USA). Samples were boiled for 5 min in Laemmli's buffer and run on Bis‐Tris gels. After electrophoresis, proteins were transferred to nitrocellulose membranes using the iBlot^®^ 2 Dry blotting system (Life Technologies, Waltham, Massachusetts, USA) and then have been blocked in 5% bovine serum albumin (BSA, Sigma‐Aldrich, St. Louis, Missouri, USA) in 0.05% TBS‐Tween (TBS‐T) for 1 h at room temperature. Next, membranes have been incubated with appropriate primary antibodies (SIRT1 (Abcam, ab12193, Cambridge, Massachusetts, USA), pyruvate kinase (Abcam, ab38237, Cambridge, Massachusetts, USA), SIRT2 (Cell Signaling Technology, 2313, Danvers, Massachusetts, USA), and γ‐tubulin (clone GTU‐88, Sigma, T6557)) in 5% BSA in 0.05% TBS‐T overnight at 4 °C. Following incubation with diluted secondary antibodies (anti‐mouse IgG linked to horseradish peroxidase (DAKO, P0447, Santa Clara, California, USA), anti‐rabbit IgG linked to horseradish peroxidase (DAKO, P0448, Santa Clara, California, USA) in 0.05% TBS‐T for 1 h at room temperature, proteins were visualized with an enhanced chemiluminescence Western blot detection system (GE Healthcare Bio‐Sciences AB using the ChemiDoc™ Touch Gel Imaging System (Bio‐Rad Laboratories, Inc., Hercules, California, USA). Immunoblots were quantified using Image Lab software (Bio‐Rad Laboratories, Inc., Hercules, California, USA).

### High‐resolution respirometry

High‐resolution respirometry was performed as previously described (Morato *et al*., 2015).

### Immunohistochemistry

Spinal cords were harvested from 13‐month‐old WT and *Sirt2* ^−/−^ mice, after perfusion with PFA 4%, basically as described (Morato *et al*., 2015). Spinal cords were embedded in paraffin, and serial sections (5 μm thick) were cut in a transversal or longitudinal (1‐cm‐long) plane. The number of abnormal specific profiles was counted at every 10 sections for each stain. At least three sections corresponding to the dorsal columns of the spinal cord were analyzed per animal and per stain. The sections were stained with hematoxylin and eosin and Sudan black, or processed for immunohistochemistry using glial fibrillary acidic protein (GFAP) [Dako, rabbit polyclonal, 1:500, Santa Clara, California, USA], synaptophysin [Dako, monoclonal, 1:500, Santa Clara, California, USA], CD68 [Abcam, rabbit polyclonal, 1:500, Cambridge, Massachusetts, USA], and RT97 [Boehringer, rabbit polyclonal, 1:500]; and with Iba‐1 [Wako, rabbit polyclonal, 1:1000] used as a marker of microglial cells. The results were expressed as the mean ± standard deviation.

### Statistical analysis

The data are presented as mean ± standard deviation (SD) or standard error of the mean (SEM). Significant differences were determined by Student's *t*‐test or repeated‐measures ANOVA to measure circadian activity (*P <* *0.05, **P <* *0.01, ***P <* *0.001) after verifying normality. Statistical analyses were performed using the software program spss 12.0.

## Funding

This study was supported by grants from the Spanish Institute for Health Carlos III and ‘Fondo Europeo de Desarrollo Regional (FEDER), Union Europea, una manera de hacer Europa’ [FIS PI11/01043, FIS PI14/00410], the Autonomous Government of Catalonia [SGR 2014SGR1430] to A.P., The Spanish Institute for Health Carlos III and ‘Fondo Europeo de Desarrollo Regional (FEDER), Union Europea, una manera de hacer Europa’ [Miguel Servet program CP11/00080, CPII16/00016, FIS PI15/00857] to S.F. and the Center for Biomedical Research on Rare Diseases (CIBERER) to M.R., and the Spanish Institute for Health Carlos III and FEDER funds from European Union (‘A way to build Europe’) [FIS grants PI14/01115, PI13/00584, and PI14/00328], Foundation La Marató de TV3 [345/C/2014], and the Autonomous Government of Catalonia [2014SGR168] to M.J., M.P.O., and R.P.

The studies conducted at the Chromatin Biology Laboratory were supported by the Spanish Ministry of Economy and Competitiveness‐MINECO [SAF2011‐25860, SAF2014‐55964R] to A.V.

## Author contributions

AP and SF conceived and coordinated the project; SF, LM, JP, MR, TRC, MJ, AN, and PMR performed experiments; SF, LM, JP, MR, TRC, MJ, AN, PMR, MD, IF, FV, RP, AV, MPO, and AP analyzed and interpreted data; FV, RP, AV, and MPO revised the manuscript; and SF and AP wrote and revised the manuscript.

## Conflict of interest

The authors declare that this article was conducted in the absence of any commercial or financial relationships that could be construed as a potential conflict of interest.

## Supporting information


**Fig. S1** Circadian Activity in aged *Sirt2* ^−/−^ mice.
**Fig. S2** Muscle tone, pain sensitivity and emotional reactivity in aged *Sirt2* ^−/−^ mice.
**Fig. S3** No cognitive deficit in aged *Sirt2* ^−/−^ mice.
