# Supplementary material for: Loss of SIRT2 leads to axonal degeneration and locomotor disability associated with redox and energy imbalance
Source: Aging Cell. 2017 Oct 5;16(6):1404–13. doi: 10.1111/acel.12682 (PMC5676070; doi:10.1111/acel.12682)
Supplement: Supplementary file 1 — Fig. S1 Circadian Activity in aged Sirt2 −/− mice. Fig. S2 Muscle tone, pain sensitivity and emotional reactivity in aged Sirt2 −/− mice. Fig. S3 No cognitive deficit in aged Sirt2 −/− mice. [file ACEL-16-1404-s001.pdf]

Circadian Activity

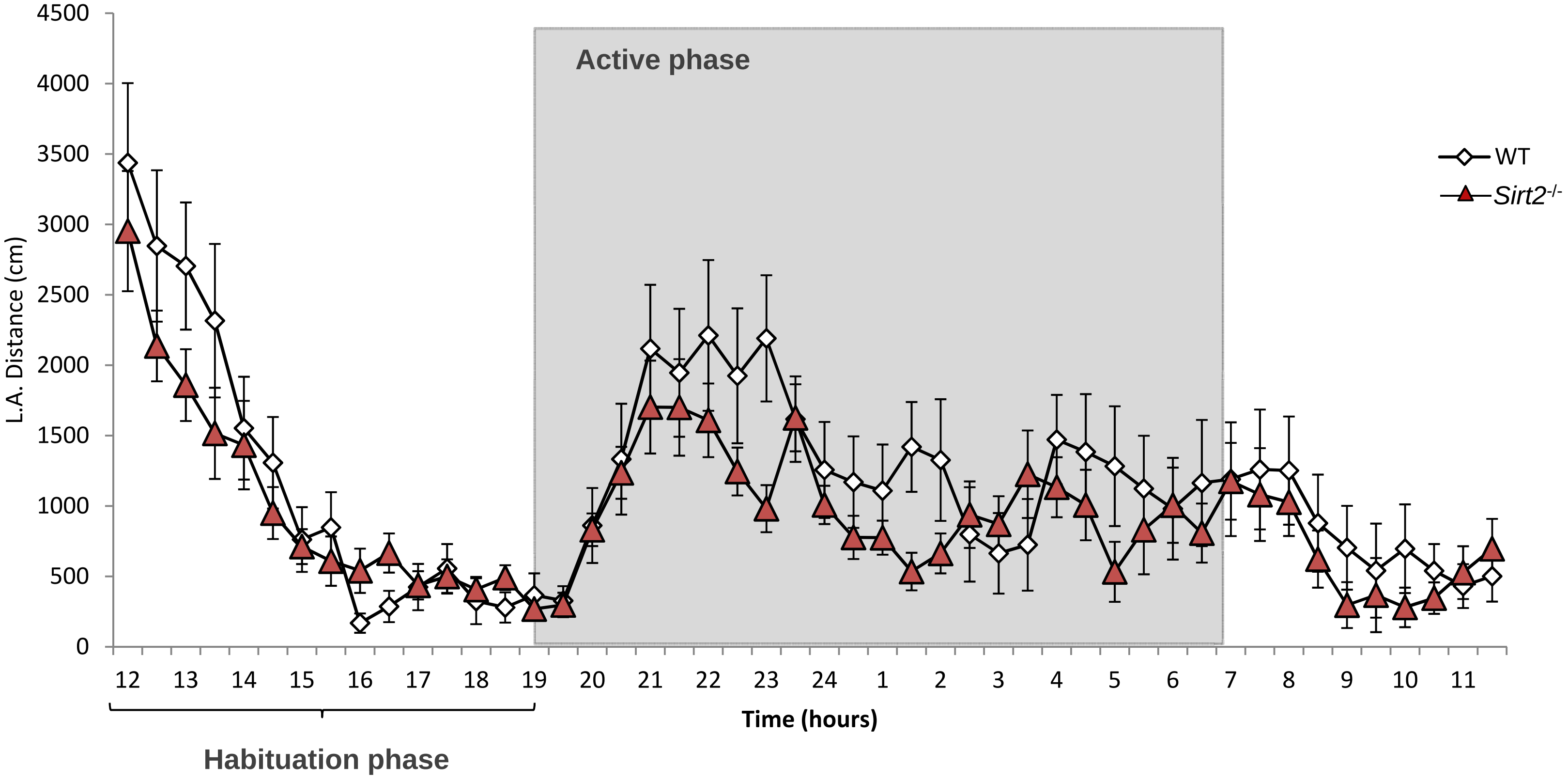

Supplemental figure 1

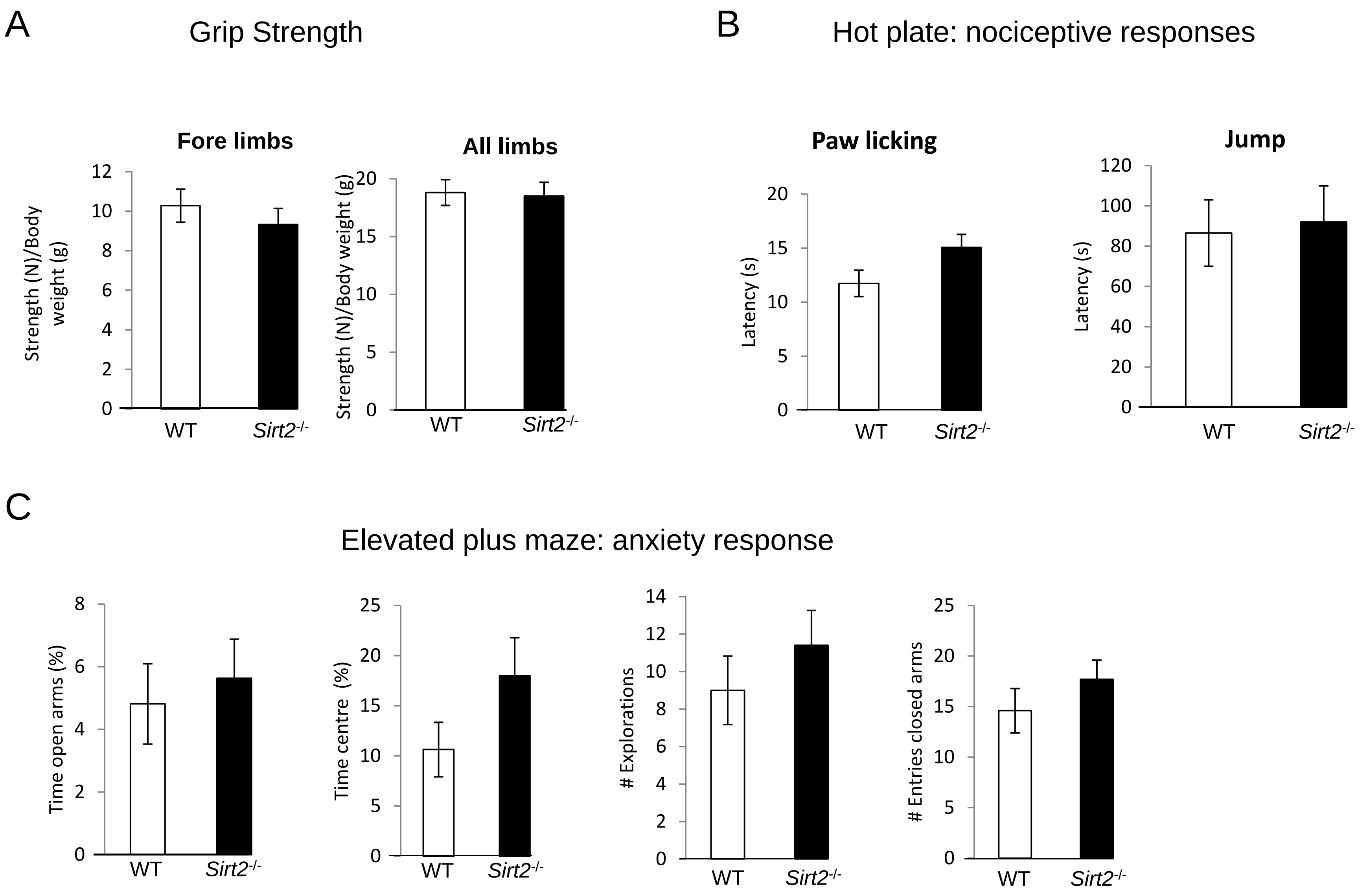

Supplemental figure 2

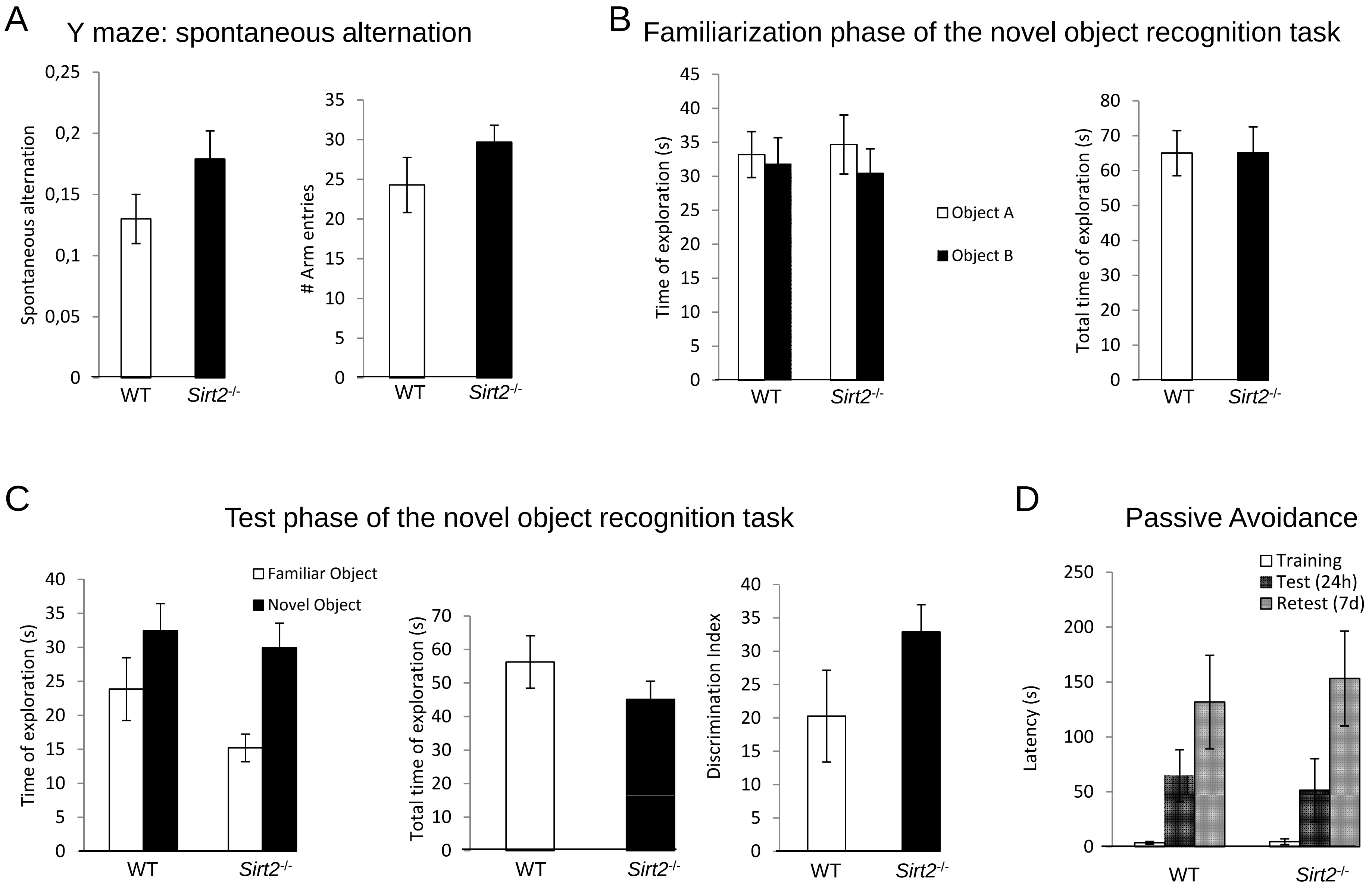

Supplemental figure 3
